# Unraveling *Neisseria meningitidis* pathogenesis: from functional genomics to experimental models

**DOI:** 10.12688/f1000research.11279.1

**Published:** 2017-07-26

**Authors:** Marco Soriani

**Affiliations:** 1 Toscana Life Sciences Foundation, Sienna, Italy

**Keywords:** Neisseria meningitidis, N. meningitidis, pathogenesis, genomics, omics

## Abstract

*Neisseria meningitidis* is a harmless commensal bacterium finely adapted to humans. Unfortunately, under “privileged” conditions, it adopts a “devious” lifestyle leading to uncontrolled behavior characterized by the unleashing of molecular weapons causing potentially lethal disease such as sepsis and acute meningitis. Indeed, despite the lack of a classic repertoire of virulence genes in *N. meningitidis* separating commensal from invasive strains, molecular epidemiology and functional genomics studies suggest that carriage and invasive strains belong to genetically distinct populations characterized by an exclusive pathogenic potential. In the last few years, “omics” technologies have helped scientists to unwrap the framework drawn by *N. meningitidis* during different stages of colonization and disease. However, this scenario is still incomplete and would benefit from the implementation of physiological tissue models for the reproduction of mucosal and systemic interactions *in vitro*. These emerging technologies supported by recent advances in the world of stem cell biology hold the promise for a further understanding of *N. meningitidis *pathogenesis.

## Introduction

*Neisseria meningitidis* is a versatile organism capable of adapting to the different environments it encounters during colonization and invasive disease. Like many other bacterial pathogens, it finds it beneficial to keep the host alive to allow transmission. However, it is a fact that in crowded settings such as military camps, universities, and schools, *N. meningitidis* tends to become more virulent ^1^. Whether this is related to the chance to encounter more appropriate environmental conditions (for example, weakened immunity, affordable nutrients, and reduced niche competition) or to the fact that, under low population density, selection pressure would keep the host alive until transmission is possible is still indefinite. Nevertheless, household contacts of patients with meningococcal disease have been shown to be at increased risk of meningococcal carriage and disease. From a genomic perspective, *N. meningitidis* is a highly diverse species, undergoing frequent recombination characterized by horizontal gene transfer ^2^. However, phylogenetic and genealogical analyses have revealed the presence of a limited number of clonal complexes associated with invasive disease (often referred to as “hyper-invasive lineages”) ^2^. These lineages show a recurrent antigenic and disease phenotype and have been an important paradigm for designing intervention strategies. The advent of “next-generation” sequencing has revolutionized the molecular epidemiology field by offering the opportunity of a complete picture of *N. meningitidis* genotypes and improving our understanding of meningococcal pathogenesis (for an in depth review on recent advances in population genomics, see 3). In this context, initiatives such as the Meningitis Research Foundation meningococcus genome library ( http://www.meningitis.org/research/genome) are expected to facilitate not only population genomics approaches but also functional genomics by guiding the selection of the most appropriate isolates and reduce the use of often irrelevant laboratory strains. An interesting application of this tool has been in the vaccine field, where this library has been instrumental in establishing that a recent rise in serogroup W cases since 2009 belongs to ST-11, a particularly virulent sequence type with a high case fatality rate ^4^.

For years, the specificity of *N. meningitidis* for humans has been the main bottleneck in unravelling the mechanisms beyond its invasive behavior. In particular, the lack of appropriate animal models resembling the clinical presentations of the human disease has affected the capacity to develop efficacious preventive interventions. In the last decade, molecular and structural evidence has highlighted a number of surface molecules with a strong specificity for human serum factors. In particular, factor H-binding protein (fHbp) has been at the center of great interest not only for its role in *N. meningitidis* pathogenesis ^5^ but also for its capacity to generate strong bactericidal antibodies after immunization in humans ^6^. fHbp is currently one of the components of the recently approved vaccines against type B meningococcus and likely to contribute to the extraordinary data on the efficacy of serogroup B meningococcal vaccine in the UK ^7^. Serogroup B is now the most common cause of outbreak-associated disease, and the fact that the novel, multi-component, protein-based Bexsero™ vaccine turned out to be 82.9% effective after two doses in preventing serogroup B *N. meningitidis* disease in British infants younger than 12 months of age ^7^ turns a promise into reality. However, the success of the strategy, as for that of all vaccines, will depend on the breadth of implementation and the promptness of the pathogen to epidemiologically adapt to the evolutionary pressure introduced by vaccination campaigns. Therefore, whatever would be the most optimistic scenario, it is important to continue to monitor, investigate, and consider all of the subtle strategies beyond the peculiar habit of this “smart” microorganism disguised as a commensal but with the license to kill. Several scientists refer to these events as an “accidental lethality” or “pathogenic commensalism”. In this commentary, we will go through the salient steps of *N. meningitidis* pathogenesis that, thanks to the support of “omics” technologies and advanced infection models, have been fully unraveled in the last decade.

## Disclosure of *Neisseria meningitides* pathogenesis by “omics” and experimental models

**Figure 1.**
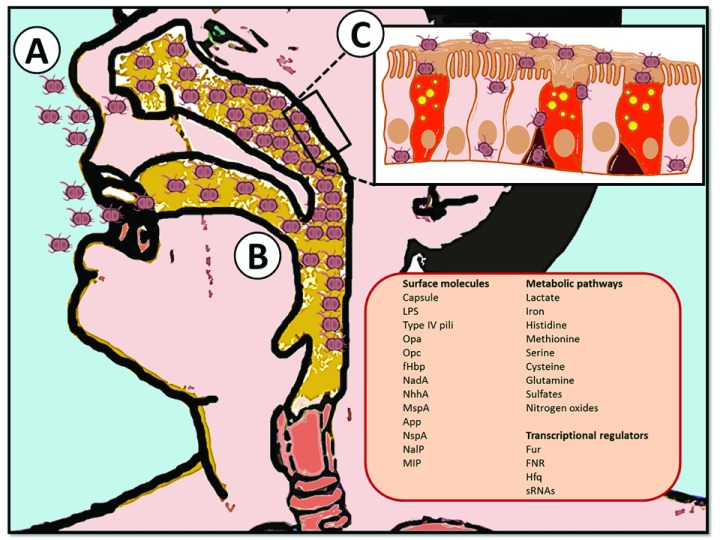
From colonization to dissemination: graphical representation of *Neisseria meningitidis* pathogenesis. ( **A**) *N. meningitidis* is spread by exchanging respiratory and throat secretions during close contacts between individuals. ( **B**) It then gets access to the nasopharynx, where it adheres to the mucosae of the mucociliary epithelium and resides as a commensal microorganism until environmental conditions are suitable for dissemination. ( **C**) Crossing of the mucosal epithelial barrier occurs by intra- or inter-cellular routes allowing entry into the bloodstream, where it quickly proliferates. This event causes sepsis and eventually (after translocation of a further physiological barrier such as the blood-brain barrier) meningitis. The right bottom panel is a list of factors/pathways defined by functional genomics studies to be determinant during various steps of *N. meningitidis* pathogenesis such as colonization of the nasopharynx and survival in blood. App, adhesion and penetration protein; fHbp, factor H-binding protein; FNR, fumarate and nitrate reductase regulator protein; Fur, ferric uptake regulation protein; Hfq, cofactor RNA-binding protein; LPS, lipopolysaccharide; MIP, macrophage infectivity potentiator MspA, meningococcal serine protease A; NadA, *Neisseria* adhesin A; NalP, *Neisseria* autotransporter lipoprotein; NhhA, *Neisseria* hia homologue A; NspA, Neisserial surface protein A; Opa, opacity protein; Opc, opacity protein C; sRNA, small non-coding RNA.

*N. meningitidis* usually resides in the human nasopharynx where it spends most of its life as a commensal microorganism by exploiting nutrients present on the mucosae ^8, 9^. Notably, Veyrier *et al*. ^10^ recently postulated that cell shape evolution of *N. meningitidis* (from bacillus to coccus) has allowed an increased adaptation to the nasopharynx by reducing the cell surface sensible to immune attacks through the modification of the peptidoglycan and by redistributing surface determinants such as pili ^10^. The initial steps of colonization and pathogenesis are graphically summarized in Figure 1, in which the emphasis is on the factors that have been identified so far as essential for *N. meningitidis* “sojourn” in the host.

Crossing of the epithelial cell layer of the nasopharynx is a rare event but, when it occurs, leads to invasion of the bloodstream, where bacteria are capable of eluding the immune system and of reaching the meninges. The ability of *N. meningitidis* to bind to ligands present on the surface of host cells allows the bacterium to easily enter in contact with the endothelial cell layer of the brain vessels and to form microcolonies ^11^. This interaction, mediated mainly by the type IV pili, modulates the endothelial cytoskeleton leading to the formation of docking structures similar to the ones elicited by leukocytes during extravasation and the consequent opening of the intercellular junctions ^12^. The sterility of the cerebrospinal fluid of the subarachnoidal space and its low serum protein content but richness in nutrients (including glucose, sodium chloride, and urea) greatly favor the replication of *N. meningitidis* and its dissemination throughout the meninges ^13^. Another phenomenon linked to *N. meningitidis* invasive disease is a generalized sepsis, in which bacteria associated with microvessels induce extensive thrombosis, coagulation, congestion, and vascular leak, leading to an extensive necrosis of the skin and surrounding tissues ^14^.

Many of the characters playing a pivotal role in this drama have recently been disclosed by the application of “omics” technologies to a number of experimental models mimicking different steps of *N. meningitidis* pathogenesis. Functional genomics, by linking genotype to phenotype, have allowed study of the correlation between gene transcript abundance or deficiency and the capacity of *N. meningitidis* to behave under various physiological conditions of the host. The first example of functional genomics in *N. meningitidis* was reported by Tang’s group almost 20 years ago ^15^. By genome-wide signature-tagged mutagenesis (STM), 73 genes essential to bacteremia were identified in an infant rat model ^15^. A few years later, with the advent of the microarray technology, new studies focused on the transcriptional events occurring during the interaction of N *. meningitidis* with host cells ^16– 19^. Then comparative genomics ^20– 23^, *in vitro* ^24, 25^ and *ex vivo* ^26, 27^ transcriptomics, proteomics ^28, 29^, and further STM ^30, 31^ completed the picture.

The scenario derived by these studies (intuitively represented in Figure 1) offers a number of considerations. As expected, adhesion molecules (such as type IV pili) and serum resistance factors (like the lipooligosaccharide and genes involved in the synthesis of the polysialic acid capsule) turned out to be essential to preserve the fitness of the bacterium under stress conditions or only to maintain its “colonizer” status. These molecules, by sensing the external milieu, need to rapidly respond to changes, whether this means the proximity to host cell ligands, the interaction with serum factors, or the availability of nutrients. This substantial surface remodeling has been exploited to identify putative vaccine candidates, as the augmented expression of surface antigens under physiological conditions has been considered a discriminating factor for selection ^16, 17, 19^. However, the dynamics ongoing during the adaptation of *N. meningitidis* to the host are far more complex and pivotal to maintain bacterial fitness. Indeed, the most intriguing results generated from functional genomics studies were relative to the modulation of genes involved in regulatory functions and metabolism. (For in-depth reviews, see 32 and 33, respectively.) It is not by chance that genomic regions coding for metabolic functions exhibit high rates of recombination ^22, 23^, a feature shared with genes contributing to pathogenicity. On the other hand, 35 of the 73 genes reported by Sun *et al*. as “essential” to *in vivo* bacteremia encode for enzymes involved in metabolism and transport of nutrients ^15^. This trend was further corroborated by *ex vivo* transcriptomic data showing that *N. meningitidis* grown in human blood differentially expresses several genes involved in nutrient transport and central metabolism ^26, 27^. Overall, transcriptomic studies have highlighted that differential expressions of genes involved in metabolism of lactate, oxidative stress response, glutathione metabolism, and denitrification pathways are among the most frequent examples of adaptive response during pathogenesis. In particular, the capacity of *N. meningitidis* to promptly catabolize lactate has been considered fundamental to bacterial survival. Lactate is broadly present in the human body at considerable concentrations (approximately 0.3 to 1.3 mM). Being a substrate for the synthesis of N-acetyl-neuraminic acids via the N-acetyl-neuraminic acid synthase (NeuB) synthetic enzyme, lactate contributes to enhanced serum resistance ^34– 36^ and nasopharyngeal colonization ^37, 38^. The evidence that, in human blood, lactate permease was significantly upregulated ^26^ further confirmed the importance of this sugar in immune evasion. However, whether the reported phenotypic behavior may act as a paradigm for *N. meningitidis* increased colonization of the nasopharynx is not clear. Indeed, an increased synthesis of sialic acid by enhancing capsule levels and lipopolysaccharide sialylation may result in an impaired ability to bind to mucosal surface. Therefore, the balance between carriage and invasive attitude is quite arguable, and more data are needed to understand the contribution of metabolic and virulence factors to *N. meningitidis* pathogenesis. The evolutionary success of *N. meningitidis* relies on an efficient replication within the bloodstream because of not only an efficacious uptake of nutrients but also the concomitant ability to evade the innate and acquired immune defenses by exploiting the benefits of an appropriate sugar decoration ^39^. Iron metabolism is also central to the fitness and ability of *N. meningitidis* to out-compete neighborhood bacteria and host defenses. Although iron is pivotal for DNA replication, electron transfer in the respiratory chain, and oxidative metabolism, free iron is scarcely available in the host and meningococci possess several iron uptake systems ^40^. Acquisition of iron from host complexes is mediated by surface-located receptors: two hemoglobin receptors (HmbR and the heterodimeric HpuAB complex) and TbpBA and LbpBA reported to bind iron-loaded transferrin and lactoferrin, respectively. However, although iron uptake is essential to *N. meningitidis* immune evasion, HmbR was recently suggested not to be required during the early stages of disease, calling into question the importance of hemoglobin in meningococcal pathogenesis ^41^. Microarray analysis of the effect of iron addition to *N. meningitidis* culture revealed a large modulation of genes involved in energy metabolism, protein synthesis, and cell envelope assembly ^42^. These events appear to be largely under the control of the ferric uptake regulation protein (Fur) regulator that, in response to iron, affects the expression of target genes ^42– 44^. For example, since the mucosal surface is rich in lactoferrin and the bloodstream contains high amounts of hemoglobin, these proteins were suggested to serve as niche indicators for *N. meningitidis*, leading to specific changes in gene expression ^42, 45^.

Environmental oxygen levels represent another important stress event encountered by *N. meningitidis* during pathogenesis. An in-depth analysis of the importance of FNR (fumarate and nitrate reductase regulator protein) in sensing oxygen concentrations was reported by Bartolini *et al*. ^46^, who elucidated a number of metabolic pathways modulated under limited oxygen conditions, as faced in the brain microcirculation.

As mentioned previously, regulatory functions are currently the hot topic in functional genomics, especially after the discovery of small non-coding RNAs (sRNAs) ^47, 48^. In *N. meningitidis*, a great deal of importance has been given to Hfq, an RNA binding protein contributing to base pairing between sRNA and mRNA ^49, 50^, found to be modulated in blood ^26^ and essential for serum resistance ^15^. A number of transcriptomic and proteomic studies confirmed the relevance of Hfq in *Neisseria* response to stress conditions ^49, 51– 53^ and its capacity to modulate sRNAs ^54^. Of importance, Capel *et al*., by exploiting a Tn-seq strategy coupled to high-throughput DNA sequencing technologies, reported a comprehensive analysis of sRNAs essential to colonize epithelial cells and primary brain endothelial cells, providing a new tool to further investigate meningococcal pathogenesis in different environments ^55^.

Unfortunately, our understanding of the pathways activated by *N. meninigitidis* in response to environmental changes is limited by the relevant number of functionally unknown open reading frames that have often been reported among the most modulated targets. To this end, Exley *et al*. found that six out of eight mutants attenuated for their capacity to adhere to nasopharyngeal explants had transposon insertions in genes of unknown function ^30^. Currently, this major gap still keeps the whole picture incomplete. It is important to notice that much of the reported evidence on the contribution of meningococcal “armaments” to adaptation and virulence was obtained by employing laboratory isolates often belonging to rare genotypes or not relevant to *N. meningitidis* pathogenesis. This is to highlight that we may still underestimate the impact of “hidden” pathways relevant to hyper-virulent lineages associated with outbreaks.

*In vitro* transcriptomic and mutagenesis studies were mainly carried out by incubating bacteria in the presence of immortalized human cell lines derived from epithelial and endothelial tissues. Although they have been a remarkable pioneering attempt to resemble human physiology of the upper respiratory tract and microcirculation, these *in vitro* studies were limited by the specificity of the events triggered by *N. meningitidis in vivo*. Recent studies on mucosal pathogens have revealed the fundamental contribution of mucosae components in triggering signals to host tissues. Nevertheless, the human specificity of this bacterium makes studying the pathogenesis of *Neisseria* infections *in vivo* very difficult. Seminal *in vitro* studies were characterized by the use of cell lines derived from organs relevant to the meningococcal disease, such as the respiratory epithelium and the brain endothelium ^56– 59^. Although the results of these studies have been pivotal to the understanding of *N. meningitidis* pathogenesis, they were limited by the lack of environmental attributes that contribute to the *in vivo* response of the host to pathogens. Experimental models of fulminant meningococcemia in human skin-grafted immune-compromised mice have recently been engineered ^60, 61^. Under these conditions, *N. meningitidis* adheres to implanted human vessels, triggering extensive vascular damage, similar to that observed in patients ^62^. We expect that this kind of model, together with the increased accessibility to organoids and three-dimensional (3D) bioprinted organs, will be extensively exploited not only to confirm the current knowledge on *N. meningitidis* pathogenesis but to disclose the hidden pathways that are essential to bacterial fitness and that could be unraveled only by recreating a physiological environment. In this context, Deosarkar *et al*. reported the first dynamic *in vitro* neonatal blood-brain barrier on a chip closely mimicking the *in vivo* micro-environment ^63^. On the other hand, models for skin, bronchi, blood vessels, and microcirculation are widely engineered for all sorts of different applications from basic research to drug discovery (nicely reviewed in 64). We therefore foresee the adaptation of 3D cellular models in novel multi-organ systems to study *N. meningitidis* pathogenesis, as has extensively been done for intestinal and gastric organoids to study enteric and *Helicobacter pylori* infections, respectively. In this context, Marrazzo *et al*. ^65^ recently established an *in vitro* 3D system which recapitulates the human tracheo-bronchial mucosa comprehensive of the pseudostratified epithelium and the underlying stromal tissue. This model has been exploited to study initial colonization events triggered by non-typeable *Haemophilus influenzae* but could easily be adapted to any other microorganism colonizing the nasopharynx. Therefore, only by stemming from the field of regenerative medicine, we could find the right approaches to unravel unknown signaling pathways occurring during *N. meningitidis* pathogenesis. Researchers working in the field of cancer progression or environmental damage to respiratory organs are generating sophisticated examples of human airways that scientists working in the infectious disease world should start considering. Table 1 is a list of 3D tissue models that have mainly been developed to study organ physiology but that could be customized to carry out studies on the strategies used by *N. meningitidis* to adapt, colonize, and induce disease in humans.

**Table 1.** List of *in vitro* three-dimensional tissue models that could be exploited in studying *Neisseria meningitidis* colonization and pathogenesis.

Nasopharynx	
*Reference*	*Synopsis*
Marrazzo *et al*. ^65^, PLOS ONE, 2016	3D reconstruction of the human tracheo-bronchial mucosa comprehensive of the pseudostratified epithelium and the underlying stromal tissue as an experimental model to study upper respiratory tract infections
Kuehn *et al*., JOVE, 2015 ^66^	Culture of the organotypic tissue bronchial and nasal culture model to study the impact of cigarette smoke on airway biology
Steinke *et al*., Biomaterials, 2014 ^67^	An engineered 3D human airway mucosa model based on a small intestine submucosa to investigate interrelations of *Bordetella pertussis* with human airway mucosa
Harrington *et al*., Molecular Pharmaceutics, 2014 ^68^	Exploitation of biomimetic porous electrospun scaffolds to develop an immunocompetent 3D model of the human respiratory tract comprised of three key cell types present in upper airway epithelium
Nguyen Hoang *et al*., Am J Physiol Lung Cell Mol Physiol, 2012 ^69^	Development of a method to generate a 3D organotypic model of the human airway mucosa in which dendritic cells are implanted
Pageau *et al*., Biomaterials, 2011 ^70^	3D *in vitro* model of the human airway that mimics bronchial morphology and function to study epithelial-mesenchymal interactions
Choe *et al*., Nature Protocols, 2006 ^71^	Human bronchial mucosal model, including a well-differentiated epithelium with functional cilia, mucus secretion, and sub-epithelial fibroblasts
Paquette *et al*., European Cells and Materials, 2004 ^72^	Tissue-engineered human bronchial equivalents from biopsies of asthmatic and non-asthmatic volunteers
Choe *et al*., American Journal of Physiology-Lung Cellular Molecular Physiology, 2003 ^73^	Tissue culture model of the human airway wall that can be induced to undergo matrix remodeling in a relevant 3D inflammatory context
Paquette *et al*., In Vitro Cellular & Developmental Biology – Animal, 2003 ^74^	Production of tissue-engineered 3D human bronchial models at the air-liquid interface
Chakir *et al*., Journal of Allergy and Clinical Immunology, 2001 ^75^	To evaluate the feasibility of an engineered human bronchial mucosa as a model to study cellular interactions in asthma
**Blood-brain barrier (BBB)**	
***Reference***	***Synopsis***
Phan *et al*., Exp Biol Med 2017 ^76^	Extensive review of microphysiological systems capturing the complexity of the blood–central nervous system interface and resembling the BBB
Wang *et al*., Biotechnol Bioeng, 2017 ^77^	Development of a microfluidic BBB model by deriving brain microvascular endothelial cells from human-induced pluripotent stem cells and co-culturing them with rat primary astrocytes on the two sides of a porous membrane
Herland *et al*., PLOS ONE, 2016 ^78^	Micro-engineering of a 3D model of the human BBB within a microfluidic chip by creating a cylindrical collagen gel containing a central hollow lumen inside a microchannel
Cho *et al*., Scientific reports, 2015 ^79^	Construction of a 3D model of BBB on a microfluidic platform
Brown *et al*., Biomicrofluidics, 2015 ^80^	Development of a microfluidic device comprised of a vascular chamber and a brain chamber separated by a porous membrane mimicking the BBB. This model allows cell-to-cell communication between endothelial cells, astrocytes, and pericytes.
Deosarkar *et al*., PLOS ONE, 2015 ^63^	Development of a BBB on a chip comprising a tissue compartment and vascular channels placed side-by-side mimicking the 3D morphology, size, and flow characteristics of microvessels *in vivo*
**Vasculature**	
***Reference***	***Synopsis***
Hoch *et al*., Eur J Cardiothorac Surg, 2014 ^81^	Extensive review of bioprinting of artificial blood vessels for 3D tissue engineering
Kolesky *et al*., Adv Mater 2014 ^82^	3D bioprinting method for fabricating engineered tissue constructs replete with vasculature, multiple types of cells, and extracellular matrix
Miller *et al*., Nat Mater, 2012 ^83^	Rigid 3D filament network of carbohydrate glass used as a cyto-compatible sacrificial template to generate cylindrical networks that could be lined with endothelial cells and perfused with blood under high-pressure pulsatile flow

References are reported in chronological order and grouped by organ/tissue specificity. 3D, three-dimensional.

## Closing remarks

This commentary started with the hope of a world without meningococcal meningitis thanks to the implementation of current vaccines. However, a lot is still needed to fully understand the pathophysiology of such a disease. The progress obtained so far in disclosing *N. meningitidis* pathogenesis reveals that the ample evidence for “culprits” is not sufficient to completely unravel the “murder scene”. Population and functional genomics have had a great role in defining many of the key pathways activated by *N. meningitidis* to successfully colonize our organism, but biotechnologies like *in vitro* 3D human experimental models are emerging as the new frontier to establish the appropriate environment to study bacterial pathogenesis. To this end, a multi-disciplinary approach would be vital to ensure the required progress for fighting human infections. In the last decade, the input of engineers, physics, mathematicians, and statisticians has been crucial to several biology and medicine areas (particularly in “omics” disciplines) and is expected to have even more relevance in the future. In the area of infectious diseases, they are becoming the principal interlocutors of molecular and cellular microbiologists by playing a pivotal role in designing, fabricating, miniaturizing, and validating *in vitro* tissue models to be exploited in host-pathogen interaction studies. Technology centers in Europe and the US (for example, the Francis Crick Institute, London, UK, and Wake Forest Institute, Winston-Salem, NC, USA) and international biotech companies (for example, Organovo, San Diego, CA, USA, and 3D Bioprinting Solutions, Moscow, Russia) are already investing in this direction by holding the promise of a future with curable diseases and a better quality of life.

## Abbreviations

3D, three-dimensional; fHbp, factor H-binding protein; Hfq, cofactor RNA-binding protein; sRNA, small non-coding RNA; STM, signature-tagged mutagenesis.
